# Diagnostic value of blood inflammatory markers for detection of acute appendicitis in children

**DOI:** 10.1186/1471-2482-6-15

**Published:** 2006-11-28

**Authors:** Ulrich Sack, Birgit Biereder, Tino Elouahidi, Katrin Bauer, Thomas Keller, Ralf-Bodo Tröbs

**Affiliations:** 1 Institute of Clinical Immunology and Transfusion Medicine, Medical Faculty of the University of Leipzig, Leipzig, Germany; 2 Department of Pediatric Surgery, Medical Faculty of the University of Leipzig, Leipzig, Germany; 3 Acomed Statistics, Leipzig, Germany; 4 Department of Pediatric Surgery, Ruhr-University of Bochum, Marienhospital Herne, Herne, Germany

## Abstract

**Background:**

Acute appendicitis (AA) is a common surgical problem that is associated with an acute-phase reaction. Previous studies have shown that cytokines and acute-phase proteins are activated and may serve as indicators for the severity of appendicitis. The aim of this study was to compare diagnostic value of different serum inflammatory markers in detection of phlegmonous or perforated appendicitis in children.

**Methods:**

Data were collected prospectively on 211 consecutive children. Laparotomy was performed for suspected AA for 189 patients. Patients were subdivided into groups: nonsurgical abdominal pain, early appendicitis, phlegmonous or gangrenous appendicitis, perforated appendicitis.

White blood cell count (WBC), serum C-reactive protein (CRP), interleukin-6 (IL-6), tumor necrosis factor α (TNF-α), acid α_1_-glycoprotein (α_1_GP), endotoxin, and erythrocyte sedimentation reaction (ESR) were estimated ad the time of admission. The diagnostic performance was analyzed using receiver operating characteristic (ROC) curves.

**Results:**

WBC count, CRP and IL-6 correlated significantly with the severity of appendiceal inflammation. Identification of children with severe appendicitis was supported by IL-6 or CRP but not WBC. Between IL-6 and CRP, there were no significant differences in diagnostic use.

**Conclusion:**

Laboratory results should be considered to be integrated within the clinical assessment. If used critically, CRP and IL-6 equally provide surgeons with complementary information in discerning the necessity for urgent operation.

## Background

It is generally accepted that appendectomy is the therapy of choice in children. Conservative management, as evaluated in some studies of adult patients [1] is not established for children. A delay in diagnosis of acute appendicitis (AA) is associated with increased risk of perforation and further complications. On the other hand in young children, geriatric patients, and in adolescent females, the negative appendectomy rate may be as high as 50 % [2]. Many attempts have been made to determine ways of decreasing the negative laparotomy rate after a clinical suspicion of AA. Under this background it would be very important to differentiate mild early appendicitis from nonspecific abdominal pain. However, despite complete clinical history, physical examination, and the usual laboratory studies clear decision aids for detection of early AA are lacking. Ultrasonography has been used increasingly in the past years with positive results and both high sensitivity and specificity rates [3]. Furthermore, the introduction of diagnostic laparoscopy and laparoscopic appendectomy in clinical pediatric surgical practice opened new horizons. One of the main question is, if laboratory tests are helpful to diagnose even early AA in Childhood. For a long time the main auxiliary test has been the leucocyte count. The diagnostic value of laboratory inflammatory markers has been studied in the past years with different and contradictory results, commonly in a heterogeneous population of adults and children [4].

The presented study comprises only patients of the pediatric age group and reflects in particular the pathophysiologic characteristics of this age group. It has to be pointed out, that conditions like pelvic inflammatory disease or acute cholecystitis play a diminished role during childhood whereas gastroenteritis, mesenteric lymphadenitis or non-specific terminal ileitis may simulate the symptoms of appendicitis in a significant part of patients with right iliac fossa pain.

The aim of our study is to find an inflammatory marker with predictive value for children who require appendectomy immediately. We intended to answer two main questions: Which laboratory values reflect best severity of appendicitis? Are there specific and sensitive constellations to discriminate between nonspecific right lower fossa abdominal pain and AA?

## Methods

This study was performed prospectively with 211 consecutive patients admitted for suspected AA between 1992 and 1996 at our clinic. 118 of them were boys (table 1). Included were all children between 4 and 14 years of age presenting with right lower abdominal pain highly suspicious for acute appendicitis. Patients with unspecific symptoms not suspicious to have appendicitis were excluded and commonly controlled in our outpatient department after 12 hours. None of them were operated on in other departments. On admission, all children were examined by an experienced pediatric surgeon, and according to the clinical judgment, the patients were classified as whether they need in-hospital observation or operation. 22 patients whose abdominal pain could not be attributed to any specific cause were categorized as having nonsurgical abdominal pain (NSAP, group 1). Altogether 189 patients underwent appendectomy during the study period. All removed appendices were examined histologically by routine protocol. According to the histopathological results, patients were classified into the following groups: absent or early appendicitis (mucosal ulceration, focal appendicitis [5]; group 2), phlegmonous or gangrenous appendicitis (diffuse infiltration of granulocytes or areas of necrosis extending through the wall; group 3), and perforated appendicitis (group 4). Furthermore, children of the groups 1 and 2 were subsummized as non severe cases, and groups 3 and 4 were considered together as a category "severe appendicitis" requiring immediate surgery. The mean age of the children as well as gender distribution in the investigated groups differed not significantly (table 1).

### Collection of serum samples

Following institutional guidelines the study was in accordance with the guidelines of the ethical committees of the participating hospitals. Peripheral venous blood was collected preoperatively in adequate tubes for determination parameters as shown in Tab. 2. Serum was stored at -70°C for later analysis of interleukins.

### Laboratory Analysis

The measurement of WBC count, platelets, and ESR were performed as usual in clinical laboratory. ELISAs for IL-6 and TNF-α were performed as batches by using commercial kits. Endotoxin (ET) was detected with the Limulus amebocyte lysate (LAL) test. CRP was analysed by nephelometry, and α_1_GP by radial immunodiffusion. Suppliers are shown in table 2. All methods were performed under ISO 15189 compliant conditions. Values below the detection limit were assumed as zero for statistical analyses.

### Statistical Analysis

Statistical analysis was performed by software package Medcalc 7.3.0 (Medcalc, Mariakerke, Belgium). Data were checked on normality (Shapiro-Wilk test). Because of rejection of normality nonparametric Kruskall-Wallis test was used for multiple group comparisons. In case of significance individual differences were identified with Mann Whitney's U test. All p values of < 0.05 were considered as statistically significant. Receiver operating characteristic (ROC) curves and the related areas under curve (AUC) were calculated using online provided data analysis tools from Acomed (Acomed, Leipzig, Germany). Tests for significance of AUC to be > 0.5, calculation of 95% confidence limits of the AUC and comparison of ROC curves according to DeLong [6] were done using the same package.

## Results

For the patients included in this study, WBC count, platelets, ESR, CRP, IL-6, TNF-α, α_1_-GP, and endotoxin were found as presented in table 3.

ESR, TNF-α, and α_1_-GP showed a poor or satisfactory correlation to the type of appendiceal inflammation and were excluded from further statistical analysis. Serum endotoxin level was elevated in cases with perforation. It failed to present usable data for differentiation of different forms of inflammation. Therefore, further analysis was restricted to CRP, IL-6 and WBC count.

**Figure 1:**
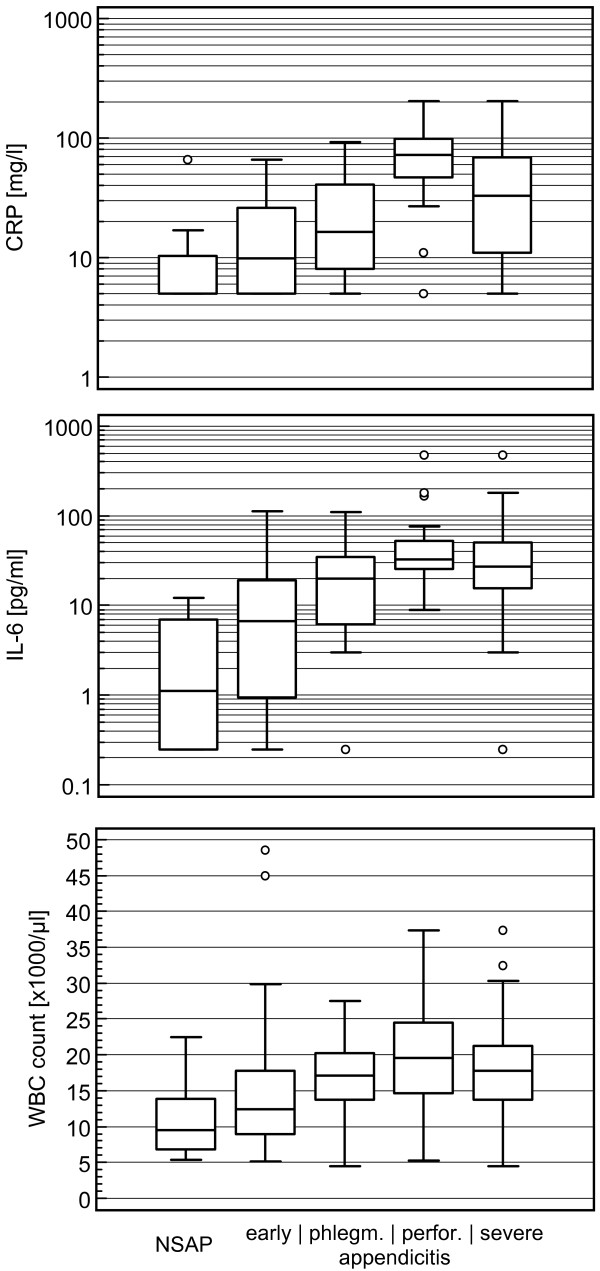
CRP, WBC count, and IL-6 show a strong correlation to the grade of local inflammation (p < 0.0001).

Significant differences between all investigated groups for CRP, IL-6, and WBC count were confirmed by the Kruskal-Wallis test (figure 1). Differences between singular groups were mostly significant (table 4). It should be noted that early appendicitis and NSAP cannot be distinguished significantly at all. However, differences between children with NSAP or early appendicitis and children with phlegmonous and perforative appendicitis (groups 3 and 4) were very impressive for all 3 parameters. Furthermore, a criterion for discrimination between phlegmonous and perforated appendicitis could be found not for leukocytes, but for IL-6 and mostly impressive for CRP.

**Figure 2:**
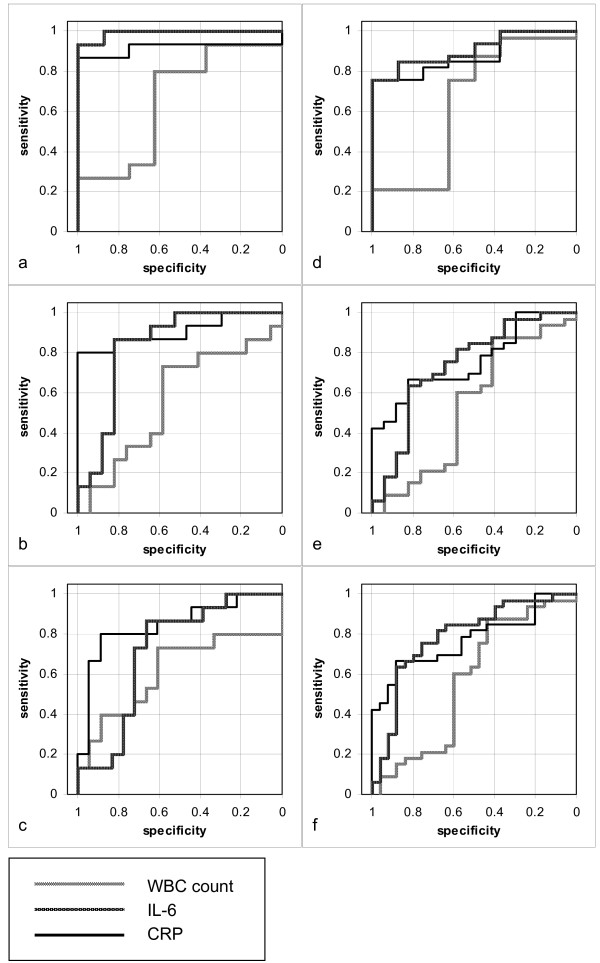
ROC curves for WBC count, CRP, and IL-6. Curves a) represent discrimination between NSAP (group 1) and perforative appendicitis (group 4), b) group 1 and cases requiring immediate surgery (groups 3 and 4), c) early appendicitis (group 2) and group 4, d) group 2 and groups 3 and 4, e) phlegmoneous (group 3) und perforative (group 4) appendicitis, and f) cases not requiring immediate surgery (groups 1 and 2) against groups 3 and 4.

To investigate diagnostic value of these parameters, ROC curves were calculated. Figure 2 depicts selected ROC curves. Table 5 represents areas under the curves (AUC) as a parameter of discrimination. Significantly best parameters are shown bold (p < 0.05 or smaller). In fact, using ROC curves non of the parameters was able to identify children with phlegmonous appendicitis (groups 3 vs. 1 and 3 vs. 2) on a statistically significant level. Measurement of IL-6 or CRP but not WBC had additional diagnostic value on the diagnosis of advanced or perforated appendicitis (groups 3 and 4). Between IL-6 and CRP, there are no significant differences in diagnostic use, but some insignificant trends as visible in ROC curves: CRP is superior when describing acuity of clinical appendicitis by better sensitivity, but IL-6 gives the best help in deciding for immediate surgery by its superior specifity.

## Discussion

Despite the diagnosis of AA will probably remain a clinical one, additional diagnostic tools are welcome. Generally a high index of suspicion is required to make the diagnosis and operate prior perforation and peritonitis. When the child exhibits the classical picture of the appendicitis syndrome, the diagnosis of acute suppurative appendicitis will generally be confirmed at operation. However many, if not the majority of patients do not present with this classical signs [7]. The results of our study support the hypothesis, that CRP, IL-6, and WBC count can help to diagnose advanced stages of AA. However, parameters highlighted by our investigation do not allow the differentiation between nonspecific abdominal pain and AA. Two distinct forms of early AA in children have been characterized by means of pathohistological methods [8].

In contrary to descriptive and comparing statistical methods, analysis of ROC curves allows estimation and verification of diagnostic suitability of diagnostic parameters. The ROC curves provide an alternative to sensitivity and specificity that allows the examination of a test's ability to discriminate between two populations regardless the cutoff level selected [9]. Therefore, we included this analysis to assess the overall diagnostic value of select parameters in clinical practice.

In the presented investigation ESR, TNF-α, and α_1_-GP showed a poor or satisfactory correlation to the type of appendiceal inflammation and were excluded from further statistical analysis for reasons of plausibility and practicability.

An increased ESR in the blood of patients with inflammatory disease can be explained by elevated concentrations of fibrinogen and other acute phase proteins [10]. It had been shown that ESR was raised in one half of children with AA and symptoms for at least 12 hours but in only one with shorter history [11]. We found a closely correlation between the ESR after one hour and the grade of inflammation. However, the method is non-specific, time consuming and not widely recommended for diagnosing rapid developing conditions such as AA.

The WBC count is the test probably most often used to support the diagnosis of AA. In the presented study the WBC count was clearly elevated in children with phlegmonous and perforated appendicitis. Differences were highly significant using the Kruskal-Wallis test. However, leucocytosis is a non-specific reaction induced by many different causes like physical stress, acute or chronic inflammation and several other conditions. This is reflected in numerous reports by an acceptable sensitivity (79–93 %) but a rather low specificity for AA [12].

The results of our study are supported by the findings, that bacteria, lipopolysaccharide (endotoxin) and other factors are known to cause an increase in the release of a number of cytokines including TNF-α, IL-1 and IL-6. IL-6, however, is one of the most potent inducer of acute phase protein synthesis in human hepatocytes. It induces the synthesis of CRP and acid α_1_-glycoprotein [10]. These changes in protein synthesis are a part of a wider response, which includes fever, high WBC count and increased immune activity. Many authors used the acute phase protein response in order to stratify the severity of disease, to evaluate the efficacy of therapy and to find out any complication.

In our study, the CRP level did not effectively predict the diagnosis of phlegmonous appendicitis, but it predicted well the patients with advanced or perforated appendicitis. Serum CRP concentration is the most widely estimated of the acute phase proteins in pediatric patients. After six to 12 hours of inflammation the concentration begins to rise and may increase a hundertfold [10]. It has been demonstrated, that in patients whose symptoms had lasted less than 24 hours WBC count had a high sensitivity while in those in whom they had lasted more than 24 hours CRP had a high sensitivity [13]. In a metaanalysis CRP has been shown to have a medium sensitivity (53–88 %) and specificy (46–82 %) for appendicitis. In children with perforated appendicitis CRP levels are significantly higher than in simple appendicitis [14]. In another pediatric study, Peltola et al. pointed out, that the predictive value of combined CRP and high WBC count was high. They regarded a negative CRP test as more informative to exclude AA than a positive one [11]. Asfar et al. recommended deferring emergency appendectomy in patients with normal pre-operative CRP to reduce the rate of unnecessary appendectomies [15]. Based on the fact that the leukocyte response is reduced in children below five years of age it was shown that the performance of WBC count was better than CRP in the correct diagnosis of AA in every age group of children except infants [4]. CRP and WBC values may be normal in 8 % of infants with proven appendicitis [4].

Between IL-6 and CRP we found no significant differences in diagnostic use.

Nevertheless, differences in ROC curves indicate that high CRP every time indicates necessity for immediate surgery. In contrast, low IL-6 reliably excludes an acute process requiring surgical intervention.

Several studies have focused on the diagnostic value of the IL-6 concentration in suspected appendicitis. Comparing with patients of a normal control group, cytokine levels of IL-1β, IL-2, IL-6, IL-8, and IL-10 were elevated in patients with AA [16]. IL-6 can be detected in patients with suspected AA, and the highest concentrations are found in patients with perforation [13,17,18]. It was concluded that in adult patients even the IL-6 concentration does not increase the diagnostic accuracy of AA, tested either ones or repeated [13,18]. Gurleyik et. al. found out, that serum IL-6 concentration was falsely negative in many patients with histologically confirmed AA [18]. But it has been shown that preoperative high increased IL-6 concentration were clearly correlated with perforation and poor postoperative conditions in adults [16-18]. We are consent with the results of [19] who investigated the diagnostic potential of IL-6, IL-8, soluble adhesion molecule CD 44, CRP, and white blood cell count to predict AA. Among these parameters IL-6 was elevated preoperatively only in gangrenous and perforated AA, and showed the best diagnostic accuracy in predicting AA [19].

Some authors believe that in the case of two or more inflammatory markers within the reference range in a patient with inconsistent clinical data, the test gives support to the policy to observe the patient closely for some time [11,13]. In a study of Paajanen [4] appendicitis was ruled out in over 90 % of cases if both leucocytes and CRP were negative.

Hallan and coworkers [12] stated, that clinical and biochemical variables must be evaluated together and used a multiple logistic regression analysis model. Adding WBC count, CRP, and neutrophil count to the clinical variables significantly reduced the perforation rate. Repeated controls of the body temperature and laboratory examinations in combination with clinical re-examinations were of benefit in the management of patients with equivocal signs of appendicitis [20]. We agree with other authors [21,22] who concluded that inflammatory variables in patients with suspected AA are of limited value and should be interpreted differently in different groups of patients. However, Lycopoulou et al. report encouraging results concerning serum amyloid A protein levels as a possible aid to discriminate between AA and nonspecific abdominal pain in children [23].

As regards biochemical tests, however, we believe that IL-6 should be more widely used than hitherto in clinical practice to exclude non-acute cases from surgery.

## Conclusion

Here, we presented the results of a pediatric study focusing on the diagnostic value of a spectrum of acute phase reactants for the diagnosis of AA. It has been shown, that WBC count, serum CRP, and IL-6 are helpful tools to support the clinical diagnosis of phlegmonous and perforated appendicitis in childhood. However, early stages of appendicitis do not strongly correlate with elevated inflammatory markes. In this cases physical examination, ultrasound investigation and clinical suspicion remain necessary to establish the correct diagnosis preoperatively.

## Competing interests

Thomas Keller owns Acomed statistics and hereby the applied ROC analysis tool. All other authors declare that they have no competing interests.

## Authors' contributions

U.S. supervised the laboratory analysis. B.B. contributed to clinical and laboratory data analysis. T.E. provided samples and data of children with appendicitis. K.B. did the cytokine examinations. T.K. analyzed the data. R.-B.T. did the design of the study together with U.S. and provided clinical samples. All authors read and approved the final manuscript.

## Pre-publication history

The pre-publication history for this paper can be accessed here:



## Figures and Tables

**Table 1:** Age, gender and number of patients included in the study

	Nonsurgical abdominal pain	Early appendicitis	Phlegmonous appendicitis	Perforated appendicitis	Total
group	1	2	3	4	
Number of patients	22	81	78	30	211
Male/female	7/15	34/47	55/23	22/8	118/93
Mean age	9.5 yr	10.7 yr	10.3 yr	9.9 yr	

**Table 2:** Laboratory examinations

Parameter	Abbreviation	Method	Reference range
White blood cell count	WBC	Hematological Counter (Beckman-Coulter, Krefeld)	4.3 – 10.8 × 10^3^/μl
Erythrocyte sedimentation rate	ESR	ESR tube (Sarstedt, Homburg)	< 10 mm/h
C-reactive protein	CRP	BN-2 (Behring, Marburg)	< 10 mg/l
Interleukin-6	IL-6	ELISA (R&D Systems, Minneapolis, U. S. A.)	< 5 pg/ml
Tumor necrosis factor-α	TNF-α		< 5 U/ml
Acid α_1_-glycoprotein	α_1_GP	Radial immunodiffusion, (Behring)	0.5 – 1.2 g/l
Endotoxin	ET	Limulus Amebocyt Lysate (Bio Whittaker, Walkersville, U. S. A.)	< 10 pg/ml

**Table 3:** Preoperative values

mean ± SD (median) [min-max]	Nonsurgical abdominal pain	Early appendicitis	Suppurative appendicitis	Perforative appendicitis
WBC count [x10^3^/μl]	11.5 ± 5.9 (9.5) [5.4–22.5]	14.5 ± 5.1 (12.6) [5.1–48.6]	17.1 ± 4.8 (17.1) [4.5–27.5]	20.3 ± 7.5 (20.2) [5.3–37.4]
ESR [mm/h]	19.3 ± 10.5 (17.0) [6.0–34.0]	29.9 ± 10.2 (22.0) [5.0–73.0]	26.2 ± 14.6 (27.0) [8.0–45.0]	60.0 ± 24.7 (57.0) [20.0–95.0]
CRP [mg/l]	10.8 ± 15.1 (5.0) [5.9–66.0]	19.9 ± 19.5 (9.9) [5.0–106.0]	28.7 ± 25.1 (17.0) [5.0–92.0]	77.7 ± 49.4 (72.0) [5.0–203.0]
IL-6 [pg/ml]	3.8 ± 4.3 (2.5) [0.0–12.2]	15.3 ± 23.2 (5.9) [0.0–112.0]	29.5 ± 34.4 (18.5) [0.0–109.8]	70.3 ± 104.9 (32.5) [9.0–472.0]
TNF alpha [U/ml]	47.4 ± 31.8 (40.0) [6.0–117.9]	81.5 ± 57.6 (40.0) [40.0–279.0]	90.3 ± 69.7 (67.5) [2.6–313.8]	138.6 ± 64.4 (147.0) [40.0–317.0]
Acid alpha1 glycoprotein [g/l]	1.0 ± 0.4 (1.0) [0.5–1.8]	1.3 ± 0.3 (1.3) [0.5–2.4]	1.3 ± 0.3 (1.3) [0.5–1.9]	1.9 ± 0.6 (1.7) [1.4–2.8]
Endotoxin [pg/ml]	13.8 ± 12.8 (6.0) [6.0–45.0]	11.8 ± 15.9 (4.5) [4.0–70.0]	12.4 ± 12.5 (4.0) [4.0–34.0]	34.7 ± 37.5 (21.0) [4.0–100.0]

significant differences are outlined in table 4.

**Table 4:** Comparison of WBC count, CRP, and IL-6 (Mann-Whitney U-Test).

WBC		1	2	3	4	3 & 4
	1		n.s.	p < 0.001	p < 0.002	p < 0.0001
	2			p < 0.0001	p < 0.001	p < 0.0001
	3				n.s.	
	1 & 2					p < 0.0001

CRP		1	2	3	4	3 & 4

	1		n.s.	p < 0.001	p < 0.0001	p < 0.0001
	2			p < 0.05	p < 0.0001	p < 0.0001
	3				p < 0.0001	
	1 & 2					p < 0.0001

IL-6		1	2	3	4	3 & 4

	1		n.s.	p < 0.001	p < 0.0001	p < 0.0001
	2			p < 0.05	p < 0.0001	p < 0.0001
	3				p < 0.02	
	1 & 2					p < 0.0001

n.s. means not significant.

**Table 5:** Best cut-off values, specifity, sensitivity, and areas under the curve (AUC) with 95 % confidence intervals estimated for CRP, IL-6 and WBC. Significantly best parameters are shown bold with italic p values in comparison to WBC.

		**Criterion**	**Sensitivity**	**95% CI**	**Specificity**	**95% CI**	**AUC**	**sign.**	**95% CI**
1 vs. 4	WBC	>14.3	80	51.9 – 95.4	62.5	24.7 – 91.0	0.667	*n.s*.	0.442 – 0.847
	IL-6	>12.2	93.3	68.0 – 98.9	100	62.9 – 100.0	**0.992**	*<.005*	0.836 – 1.000
	CRP	>11	86.7	59.5 – 98.0	100	62.9 – 100.0	**0.937**	*<.01*	0.752 – 0.992
1 vs. 3+4	WBC	>14.3	75.8	57.7 – 88.9	62.5	24.7 – 91.0	0.644	*n.s*.	0.479 – 0.787
	IL-6	>12.2	75.8	57.7 – 88.9	100	62.9 – 100.0	**0.903**	*<.01*	0.770 – 0.973
	CRP	>11	69.7	51.3 – 84.4	100	62.9 – 100.0	**0.843**	*<.05*	0.695 – 0.937
2 vs. 4	WBC	>18	73.3	44.9 – 92.0	58.8	33.0 – 81.5	0.569	*n.s*.	0.382 – 0.741
	IL-6	>21.7	86.7	59.5 – 98.0	82.4	56.6 – 96.0	**0.833**	*<.02*	0.660 – 0.940
	CRP	>42	80	51.9 – 95.4	94.1	71.2 – 99.0	**0.894**	*<.005*	0.734 – 0.974
2 vs. 3+4	WBC	>12.6	87.9	71.8 – 96.5	41.2	18.5 – 67.0	0.542	*n.s*.	0.395 – 0.684
	IL-6	>21.7	63.6	45.1 – 80.1	82.4	56.6 – 96.0	**0.736**	*<.05*	0.592 – 0.851
	CRP	>26	66.7	48.1 – 83.1	82.4	56.6 – 96.0	0.731	*n.s*.	0.587 – 0.846
3 vs. 4	WBC	>18.2	73.3	44.9 – 92.0	61.1	35.3 – 82.9	0.613	*n.s*.	0.428 – 0.777
	IL-6	>23.1	86.7	59.5 – 98.0	66.7	41.0 – 86.6	0.719	*n.s*.	0.535 – 0.860
	CRP	>46	80	51.9 – 95.4	88.9	65.2 – 98.3	**0.835**	*<.05*	0.665 – 0.940
1+2 vs. 3+4	WBC	>12.6	87.9	71.8 – 96.5	44	24.4 – 65.1	0.575	*n.s*.	0.438 – 0.703
	IL-6	>12.2	75.8	57.7 – 88.9	76	54.9 – 90.6	0.79	*n.s*.	0.663 – 0.886
	CRP	>26	60.6	42.1 – 77.1	88	68.8 – 97.3	0.767	*n.s*.	0.637 – 0.868
